# Seismic Resilience of Rural Water Supply Systems; Factor Analysis of Cases Set in Sichuan Province, China

**DOI:** 10.3389/fpubh.2022.840379

**Published:** 2022-02-22

**Authors:** Wenmei Zhou, Gretchen Kalonji, Chuan Chen, Igor Martek

**Affiliations:** ^1^The Hong Kong Polytechnic University Institute for Disaster Management and Reconstruction, Sichuan University, Chengdu, China; ^2^Business School, Sichuan University, Chengdu, China; ^3^School of Architecture and Built Environment, Deakin University, Geelong, VIC, Australia

**Keywords:** rural water supply, rural development, earthquake, factor analysis, resilience

## Abstract

The seismic resilience of water supply systems can be impacted by numerous factors, but what these factors are in the rural context of China is unknown. In this study, 41 potential influencing factors of seismic-resilience for rural water supply system (RWSS) were obtained through a literature review and semi-structured expert interview, comprising 26 general influencing factors (GFs) and 15 water supply safety influencing factors (SFs). This study verified and ranked these factors through a questionnaire survey delivered to RWSS stakeholders in Sichuan Province, China. Based on 123 valid, returned questionnaires, these factors are divided into 9 factor groups through factor analysis performed on GFs and SFs, respectively, of which “economic resilience” and “organizational resilience in disaster prevention stage” are shown to be the most important factor groups. Additionally, it found that the experience of earthquake events significantly affects the perceptions of stakeholders on the importance of certain factors. Specifically, stakeholders who have experienced an earthquake prioritize the post-earthquake resilience of the system, while those who have not experienced an earthquake prioritize the absorption capacity of the system in the disaster prevention stage. Thus, it is not appropriate to use fixed weights to evaluate the seismic resilience of RWSSs. Significantly, this outcome differs from existing findings on the resilience of Urban Water Supply Systems (UWSSs), where “technical resilience” is the key dimension. These findings can help decision-makers fully understand the factors affecting the seismic resilience of RWSSs in China, and in doing so, augment the strengthening of rural water supply.

## Introduction

**Figure 1:**
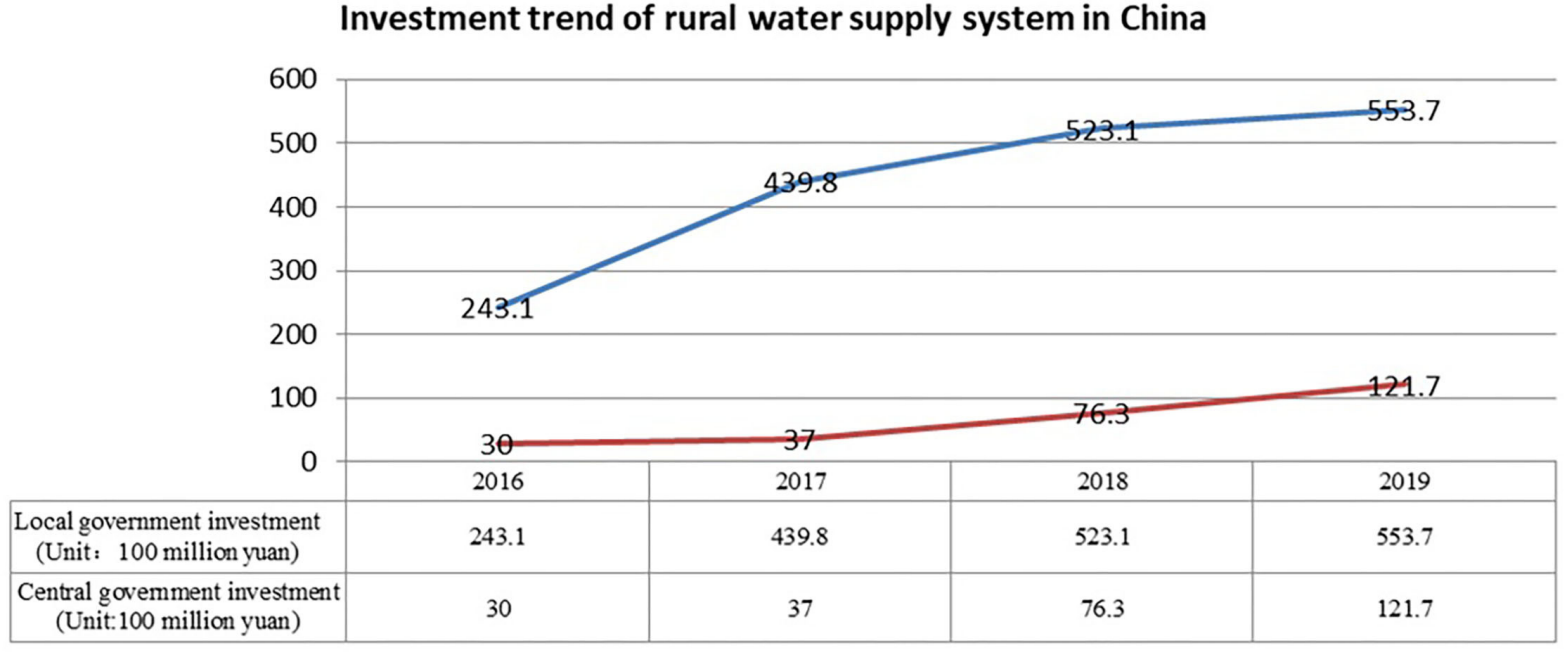
The investment trend of rural water supply systems (RWSSs) in China from 2016 to 2019 (Data sources: Ministry of water resources of China).

Access to safe drinking water is essential to human health and wellbeing. As a consequence of the successful strategies promoted by the United Nations Sustainable Development Goals, water supply services in developing countries have significantly improved, especially in rural areas (1). In China, as the government continues to increase investment in the construction of Rural Water Supply Systems (RWSSs; Figure 1), the proportion of the rural population with access to clean drinking water has risen from 68.7 to 86% over just 4 years, from 2016 to 2019. Over the same period, in urban areas, access had remained stable at about 98% (2).

However, the existence of a water supply system (WSS) in the rural areas does not guarantee that people living there have access to a sustainable and reliable supply of clean drinking water over the long term (1). In China, the majority of earthquakes occur in rural areas (3). Earthquakes in China are not only frequent but also destructive, resulting in serious damage to local WSSs and leaving large numbers of people without water supply (see Table 1).

**Table 1:** Damage to rural water supply systems (RWSSs) resulting from destructive earthquakes.

**No**.	**Magnitude**	**Date**	**Epicenter location**		**Destruction of rural water supply project (place)**	**Numbers of people left without access to water**
			**Location**	**City/suburb**		
1	7	2013/4/20	Lushan County, Sichuan	Suburb	1,727 (4)	85,000(4)
2	7.1	2010/4/14	Yushu County, Qinghai	Suburb	1,123 (5)	82,800 (5)
3	8.0	2008/5/12	Wenchuan County, Sichuan	Suburb	49,949 (6)	9,555,000

For organizations that are responsible for operating water supply infrastructure, it is vital to ensure that services are uninterrupted since water is the key factor for human survival in any disaster situation (7). It is a great challenge for the Chinese government to evaluate the seismic capacity of the rapidly developing RWSSs of China. After the occurrence of several terrorist attacks in the United States and Europe in 2000, the concept of Critical Infrastructure Protection prevails in developed countries (8–11). However, due to the inability to fully protect key infrastructure, research interest has gradually shifted from infrastructure protection to infrastructure resilience (12). Resilience in respect of natural disaster management has become the core tenet in the strategies and policies of urban planners, technical practitioners, decision-makers, and non-governmental organizations alike (13).

Given the critical infrastructure nature of WSSs, many studies have been devoted to an assessment of their seismic resilience (14). Bruneau et al. proposed a general framework, “Technical, Organizational, Social, and Economic” (TOSE), for resilience assessment of communities and infrastructure (15). Following this, many researchers have evaluated the seismic resilience of WSSs across various stages of the earthquake disaster management cycle. Chang and Shinozuka improved the ‘TOSE' model by evaluating the impact of multi-dimensional factors on the seismic resilience of WSSs in Memphis, Tennessee, USA. They developed an earthquake loss estimation model, which informs the construction of seismic capacity in the disaster prevention stage (16). Zhao et al. analyzed the seismic resilience of the urban water supply system (UWSS) at the emergency response stage, taking the water supply system of Lianyungang city in China as a case study (17). In addition, numerous studies focus on evaluating the post-disaster resilience of UWSSs through indices (18, 19) and mathematical models (20, 21). However, the existing methods of resilience quantification lack the ability to address all phases (22). Moreover, the focus of research to date has been UWSSs (23). Consequently, measuring the resilience of RWSSs to earthquakes remains effectively uncharted territory.

Resilience indicators will enable administrators at various levels to integrate resilience development strategies into mitigation and prevention plans (24). Like other phenomena, factors affecting resilience must be determined before assessing earthquake resilience (25). Therefore, the purpose of this study is to identify and rank the key factors affecting seismic resilience from the perspective of RWSS managers and to explore the way decision-makers organize these factors when evaluating the seismic resilience of RWSSs. A comprehensive list of identified and ranked factors thus provided managers of RWSSs, especially those who have not experienced destructive earthquake, with a framework against which to make better informed decisions in the practices of implementing resilience of RWSS to earthquake.

## Current Understanding of the Resilience of WSS

### The Dimensions of WSS Resilience

The concept of resilience adapted to ecological systems is defined as “*a measure of persistence of systems and of their ability to absorb change and disturbance and still maintain the same relationships between populations or state variables”* (26). Subsequently, it has been widely applied to other fields (27–29). Resilience in WSSs can be understood as a comprehensive capacity to withstand and absorb disruptions and revert quickly to the pre-disrupted state (14, 30).

The physical vulnerability of infrastructure systems has been, for decades, the dominant concern when considering the resilience to earthquake disasters (25). Liu and Song (31) summarized 21 studies on the resilience of urban water supply networks (WSNs), pointing out that researchers primarily study the seismic resilience of WSSs based on a simulation method of recovery while improving the seismic resilience of WSN from a limited, technical point of view by adding or upgrading pumps and pipeline expansions (7, 17, 32). However, Bruneau et al. found that the resilience of infrastructure systems is not limited to physical vulnerability, proposing the TOSE model for the comprehensive measurement seismic-resilience (15). The work by Bruneau et al. lays the foundation for multi-dimensional research on key infrastructure and associated communities. Researchers from different disciplines usually focus on seeking the variables of resilience when considering the seismic resilience of WSS from a multi-dimensional perspective.

Certain studies have verified the influence of specific factors on the seismic resilience of WSSs by way of mathematical models. Zhao et al. used the “recovery degree” to quantify differences in system performance pre-disaster and post-recovery (17). Through the performance response function, the recovery resources and recovery speed were verified to be the factors affecting system organization and technical resilience at the emergency response stage. Cimellaro et al. examined the case of the WSS in an Italian town situated within a seismic zone (32). They regarded the seismic resilience of water distribution networks as the product of “the number of users temporarily without water,” “the water level in the tank,” and “the water quality,” confirming the influence of technical, social, and environmental factors. Mazumder et al. analyzed the impact of environmental and technical factors (including the time to repair a break, number of breaks, network topology, the level of corrosion, and the available resources of utility companies, etc.) on the seismic resilience of water supply pipe network through probabilistic functionality fragility surface methods (33). Yoon et al. examined the impact of seismic intensity and the dependence of WSS on power facilities on the seismic resilience of urban water supply networks through a “recovery curve” (34).

Other studies have analyzed the factors affecting the seismic resilience of WSSs using actual earthquake disaster data. Mostafavi et al. conducted a qualitative study based on the Nepal earthquake in 2015 to investigate the comprehensive factors affecting the seismic resilience of WSSs in developing countries from the perspective of economic, technology, organizational, and environmental factors (35). Pribadi et al. analyzed five destructive earthquake disasters and summarized the technical and environmental factors affecting the seismic resilience of infrastructures, such as WSSs in Indonesia (36).

In addition, some studies comprehensively explored multi-dimensional potential influencing factors of seismic resilience of WSSs by means of literature reviews and expert interviews. Balaei et al. proposed the CARE model which develops the TOSE model by considering the impact of environmental factors (25). The CARE model comprises five dimensions: technical, economic, social, environmental, and organizational, along with an eight-step evaluation process. Nevertheless, it is necessary to develop indicators for each dimension in order to give the model practical effect. Based on the CARE model, Balaei et al. discussed the social impact factors (37), technical impact factors (38), and economic impact factors (39), while in subsequent research, these factors were verified using earthquake scenarios derived from New Zealand and Chile.

All these studies show that the influencing factors are the premise and basis of resilience evaluation of WSSs. Each study contributes to the evaluation of the resilience of WSSs in its own way. However, due to different research agendas, the list of all these factors and their relative importance varies considerably. At present, there is no widely and uniformly recognized list of influencing factors of the seismic resilience for WSSs.

### Spatial Differences in Resilience Research

From a geographical perspective, the scale for disaster resilience measurement is categorized into four levels: household/individual, community, national, and global (40). The most common level of seismic-resilience measurement is the community level, which is further divided into urban and rural areas (25).

In comparing the resilience across different regions at the community level, Cutter et al. (41) proposed a local-based model (disaster resilience of place, or DROP), and 36 indicators were used to analyze the seismic capacity of communities and key infrastructures across south-eastern counties of the United States. In a subsequent study, they found that there are spatial differences in resilience with the seismic resilience of urban areas being generally higher than that of rural areas (42). In addition, there are great differences in the driving factors of seismic resilience between urban and rural communities and infrastructure (42, 43).

While seismic resilience of UWSSs has attracted much attention, few researchers have considered the unique circumstances of RWSSs (14, 23). Studies highlight drinking water safety and focus on the factors influencing policies (44–47), drinking water quality (44, 47–49), and access to drinking water (50–52).

Simply, there is a large gap in the comparative knowledge of disaster resilience between urban and rural areas. Moreover, there is a lack of research on the seismic resilience of RWSSs in China. No assessment has been made as to whether the factors affecting the seismic resilience of UWSSs similarly affect the resilience of RWSSs. Neither has a relationship been established between factors affecting rural drinking water safety and RWSS resilience. Given the urgent need for development of China's RWSSs these relationships warrant investigation.

### The Role of Stakeholders in Resilience Practice

Stakeholders refer to the individuals or entities that provide input in the decision-making process and benefit from the decision-making results (53, 54). Previous studies have discussed the role of stakeholders in disaster risk management in the disaster reduction stage (55) and the impact of stakeholder attributes on post-disaster reconstruction in the disaster recovery stage (54). Research shows that stakeholders play a key role in disaster prevention, response, and recovery. Therefore, our goal is to explore the influencing factors of seismic resilience of RWSSs in China and reflect the potential influence mechanism of seismic resilience of RWSSs from the perspective of stakeholders.

A better understanding by stakeholders of the determinants of the resilience of water infrastructure systems is essential for prioritizing the allocation of limited resources in developing countries to reduce the adverse impact of natural disasters on communities (35). However, the occurrence of disasters will affect the views of stakeholders on resilience, resulting in different decisions in the implementation of resilience practice (56). Compared with other natural disasters, such as floods, earthquakes are the most destructive, but the probability of occurrence is relatively low. Therefore, not all stakeholders of RWSS have experienced earthquakes. However, in recent years, some earthquakes have occurred in non-traditional seismic zones, such as “6.17 Changning earthquake” in 2019 and “9.16 Luxian earthquake” in 2021, which implies that most RWSSs need to do a good job in earthquake prevention and disaster reduction in order to deal with possible earthquake disasters.

Therefore, according to the research status of seismic resilience that influence factors of WSS in the literature, considering the impact of earthquake occurrence on stakeholders, there are two research gaps in the research of seismic resilience of RWSSs in China. Consequently, this study has the following three objectives:

1. To identify and rank the factors affecting seismic-resilience of RWSSs in China.
2. To reveal the effects of spatial differences in determining the importance of factors.
3. To cluster the factors into groups that reflect the underlying mechanism in evaluating seismic-resilience of RWSSs.

## Materials and Methods

### Research Process

**Figure 2:**
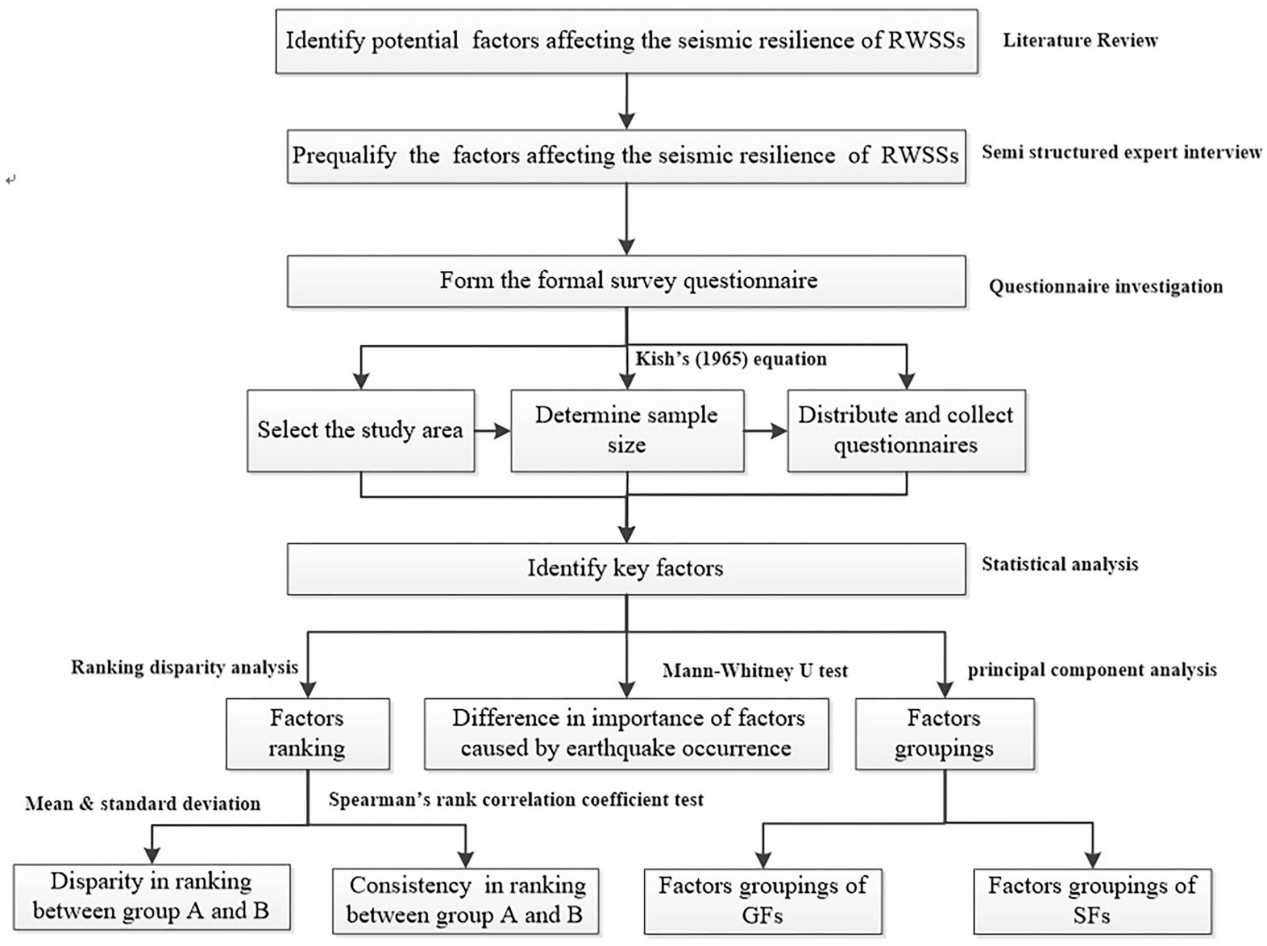
The research process of factors affecting the seismic-resilience of RWSSs.

To achieve these objectives, the research process is conducted in stages, as shown in Figure 2. As this study aims to investigate the importance of factors affecting the seismic-resilience of RWSSs and the latent relationships among them, the first step is to collect the potential influencing factors of RWSSs through literature review, which is a common method in factor studies (1, 7, 37–39, 42, 54). Then, a questionnaire survey based on the potential factors collected by literature review is suitable for data collection and is undertaken to collect the professional views of stakeholders of RWSSs. This approach is widely used and recognized by researchers in the domain of disaster management studies (54). To determine the key factors affecting the seismic resilience of RWSSs and the impact of earthquake experience on stakeholders, a series of statistical analyses were carried out on the questionnaire data by SPASS 20.0.

### Identification of Potential Factors

This study obtained a list of potential factors affecting the seismic resilience of RWSSs in China through a comprehensive literature review. The list of potential factors consists of two parts. The first part is the general influencing factors of seismic resilience (GFs), and the second part is the factors that influence rural drinking water safety (SFs). The list of GFs is determined according to the related research on influencing factors of seismic resilience considered in disaster prevention, emergency response, and post-disaster recovery of WSS, while the list of SFs is determined according to the relevant researches on rural drinking water safety, such as discussing the management of rural safe drinking water project, the rural water source environment, policy, etc. Since the factors proposed in these studies are different in names, numbers, and meanings, it is necessary to combine them into a single list of factors, which forms an initial list of influencing factors, including 40 potential general resilience influencing factors and 22 potential safe drinking water influencing factors.

The initial factors list was prequalified through a pre-assessment exercise between July and August 2020, in China. Ten experts from public, private, and research institutions with at least 5 years of experience relevant to the rural water sector and participated in at least one earthquake relief of WSS voluntarily took part in the pre-assessment phase of the study. The experts were selected in the early stages of the study from a variety of disciplines, namely, disaster resilience, WSS operations, management, the social sciences, and economists. The interviewees were selected through a consulting firm (ROCA Consulting), local authorities (Jiuzhaigou County Government, Economic and Information Bureau of Wenchuan County), Universities (Sichuan University, Deakin University, and Chengdu University of Technology), emergency management departments (Chengdu Emergency Management Bureau; Emergency Management Bureau of Jiuzhaigou County; Luzhou emergency management department), the head of a water company (the person in charge of urban and rural water supply and drainage integration in Mianzhu City), and independent experts. Semi-structured questionnaires were used to overcome biases and heuristics that can affect results. Interviewees were asked to comment on and modify the potential indicators concerning the seismic resilience of RWSSs. Specifically, (1) The redundancy indices with various descriptors used in the literature, but which describe similar items were merged. As an example, “social trust,” “trust in the government,” and “trust in rescue” are unified here and merged into “social trust.” (2) Similarly, inapplicable factors were removed. This includes terms such as “GDP,” “the universal vulnerability index,” and “the world risk index,” which cannot be applied at community levels. (3) Classifications such as GFs and SFs were added to highlight the characteristics of RWSSs and facilitate data analysis. For example, “professional reserve” and “maintenance information” have an impact on the seismic resilience of both UWSSs and RWSSs. However, in rural areas, the lack of professionals and imperfect maintenance information are more likely to become the influencing factors restricting rural seismic resilience. Thus, they are summarized here as SFs. The results were used to improve the indicators derived from previous research in order to more appropriately evaluate the seismic resilience of RWSSs.

Following the above steps, 26 GFs and 15 SFs were identified as potential factors, all of which are cited no less than 2 times, as shown in Table 2. Thus, a total of 41 comprehensive influencing factors affecting the seismic resilience of RWSSs are retrieved for further research.

**Table 2:** List of potential factors affecting seismic-resilience of RWSSs.

**No**.	**Factors**	**References**
GF01	Alternative water source	(14, 15, 25, 35, 57, 58)
GF02	Seismic design	(15, 25, 59)
GF03	Emergency Power	(14, 35, 57)
GF04	Independent Fire-water design	(15, 60)
GF05	Early warning system	(39, 61)
GF06	Remaining service capacity	(14, 15, 58)
GF07	Degree of system recovery	(15, 17, 35)
GF08	Topographic	(40, 43, 62)
GF09	Emergency response plan	(7, 25, 35, 42, 57, 58)
GF10	Community participation	(14, 38, 39, 42, 59)
GF11	Effective partnership	(7, 57, 59, 61, 63)
GF12	Leadership	(7, 59, 63)
GF13	Decision-making	(7, 63)
GF14	Emergency water supply	(7, 15, 58)
GF15	Organizational structure	(7, 15, 35, 63)
GF16	Crisis insight	(7, 37, 42, 59)
GF17	place attachment	(7, 37, 42, 59)
GF18	Social trust	(37, 59)
GF19	Post-disaster water demand	(15, 25, 35, 58)
GF20	Operation and maintenance funds	(35, 45, 61)
GF21	Available financial resources	(15, 25, 39, 58)
GF22	Fast financing access	(15, 25, 39)
GF23	Earthquake intensity	(17, 35, 64)
GF24	Earthquake history	(17, 35, 40, 64)
GF25	Reconstruction mode	(17, 65, 66)
GF26	The time of the earthquake	(25, 35, 67)
SF01	Professional reserve	(44, 47, 49, 61)
SF02	Maintenance information	(35, 61)
SF03	Household water reserve	(35, 50, 61)
SF04	Political will	(7, 51, 68)
SF05	Proactive posture	(7, 63)
SF06	Periodic asset assessment	(7, 35, 68)
SF07	Groundwater stock	(35, 47, 52)
SF08	Intelligent design	(14, 35, 47, 68)
SF09	Climate conditions	(40, 47, 68)
SF10	Laws and policies	(45, 47, 51)
SF11	Cultural level	(37, 42, 59, 61, 64)
SF12	Community publicity	(7, 15, 25, 47)
SF13	Employment rate	(7, 25, 40, 47, 57)
SF14	GRP	(47, 48, 52)
SF15	Environmental pollution	(45, 47, 48, 50)

### Questionnaire Survey

The data was collected through an online structured questionnaire that comprised three distinct sections based on the factors list (see Table 2). Section Introduction illustrates the objective along with confidentiality commitments. Section Current Understanding of the Resilience of Water Supply Systems (WSS) collects general information about respondents. Section Materials and Methods tests the importance of the 41 factors identified from literature reviews and experts as these are potentially able to affect the seismic resilience of RWSSs. The responses to most questions were on a five-point Likert scale, unless otherwise stated, where 1 and 5 represented the lowest and the highest levels of importance, respectively.

### Study Area and Questionnaire Distribution

According to the statistical data of the China seismic network, Sichuan Province is one of the most earthquake damage-prone regions in China. Similarly, Sichuan has suffered greatly from RWSSs failure as a consequence of earthquakes, such as the Wenchuan earthquake in 2008 and the Lushan earthquake in 2013. Thus, RWSSs in Sichuan Province were selected as the context for this study. Since not all RWSSs in Sichuan are susceptible to earthquake disasters, the sampling frame was filtered by focusing on 1,296 RWSSs which were located near the earthquake zone according to the list of RWSSs published by the Sichuan Provincial Water Resources Department in July 2019 (69).

To obtain a statistically representative population sample, Kish's (70) equation is used, being an established precedent set in other studies on determining sample size:


(1) $$\begin{array}{r} n=\frac{m}{1+\frac{m}{N}} \end{array}$$

In Equation (1), *N* and *n* denote the total population and the sample size from a finite population, respectively, while m represents the sample size from an infinite population, which can be calculated by:


(2) $$\begin{array}{r} m=\frac{S^{2}}{V^{2}} \end{array}$$

In Equation (2), *V* denotes the standard error of the sample population with a confidence level of 95%. V equals 0.05 and *S*^2^ = P (1-P), where *S*^2^ refers to the standard error variance of population elements, with *p* = 0.5 deemed a “safe” choice according to Kish's recommendation (70). Consequently, m is equal to 100. Based on the equations, an acceptable sample size of 93 is determined by:


(3) $$\begin{array}{r} n=m/\left(1+m/N\right)=100/\left(1+100/1296\right)=92.84\approx 93 \end{array}$$

**Figure 3:**
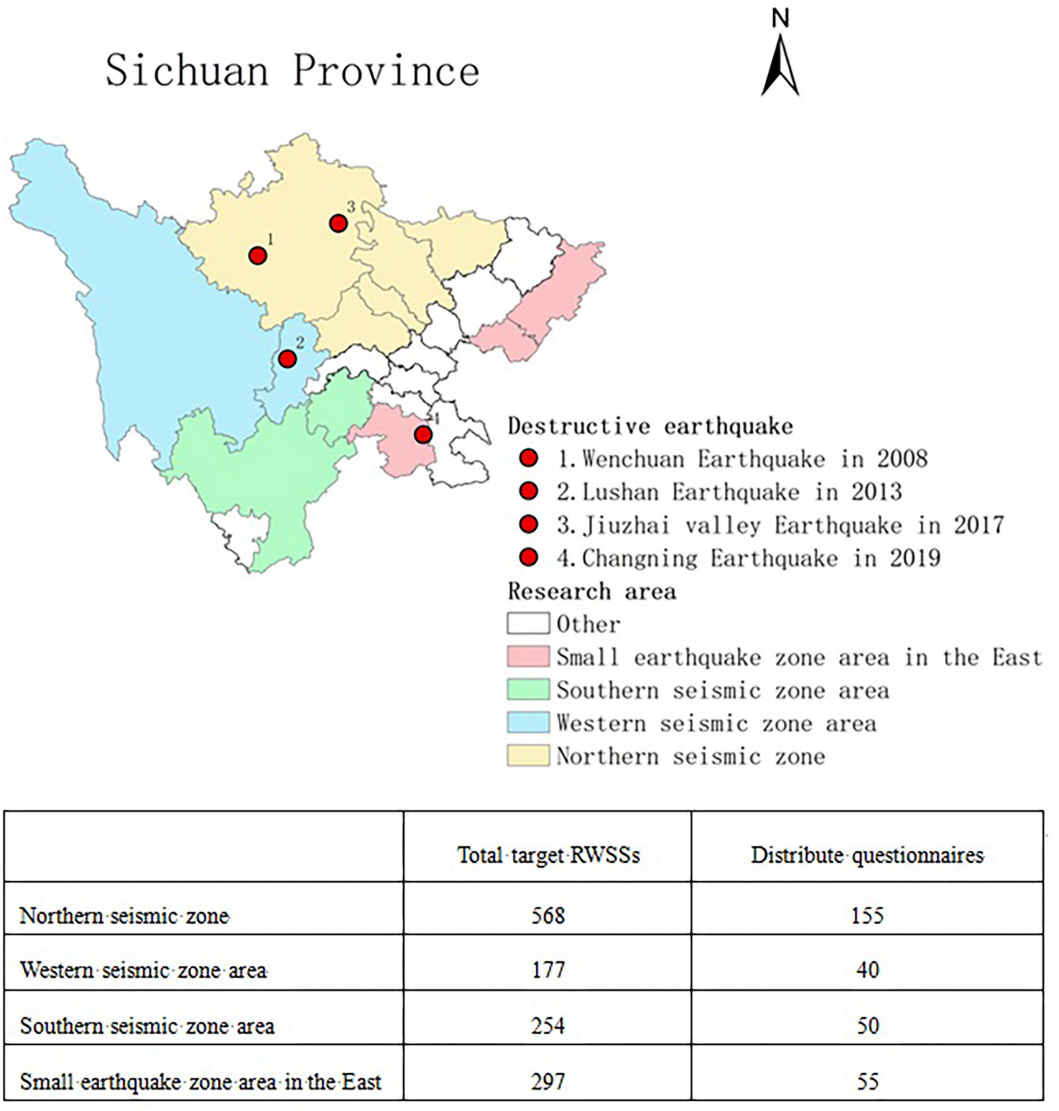
Distribution map of survey area in Sichuan Province.

In order to avoid regional bias, a careful sampling design should be carried out to obtain samples reflecting different regional characteristics contained within the study area before sampling (71). Considering the distribution characteristics of seismic zones in Sichuan Province and data availability in rural areas, according to the opinions of experts, this study divides the rural areas of Sichuan Province into four regions according to the distribution of seismic zones (see Figure 3). As a result, the revised structured network questionnaire was sent to 300 stakeholders of RWSSs by e-mail or WeChat (the most common communication platform in China) from September 2020 to February 2021. A total of 135 questionnaires were collected, of which 12 were judged to be invalid (where the importance option scores of 41 factors in 9 questionnaires were all rated 1 or 5, and where the response time of a further 3 questionnaires was significantly shorter than that of the other questionnaires, coming in at under 1 min). The remaining 123 valid questionnaires exceeded the 93 samples required for statistical validity. Thus, the questionnaires were judged representatively. Moreover, compared with similar studies in the field of disaster management (72), the 123 valid questionnaires of this study are deemed sufficient. Cronbach α's coefficient is 0.967, exceeding the recommended reliability of 0.7 (73), indicating the questionnaires are reliable.

### Data Analysis Technique

In order to capture the demographic details of the interviewees, descriptive analysis was carried out first. In addition, in order to analyze whether the occurrence of earthquake disasters has a significant impact on the judgment of respondents on the relative importance of influencing factors, the respondents were divided into two groups—Group A and Group B—according to their experience in participating in earthquake relief of RWSSs.

Considering the sample size of this study, the mean value is used for ranking and determination of the critical factors considered by Group A and Group B, rather than artificial intelligence algorithms that require a large amount of data (74). The standard deviation is used to further determine the rank order when the mean values of several factors are the same (73, 75). At the same time, in order to determine the important factors impacting seismic resilience of RWSSs, formula 4 is used to determine the threshold for dividing important factors (76). According to the calculation results of formula 4, the threshold of important factors in this study is set at 3. When the mean value of a factor is greater than 3, the factor is judged as significant; otherwise, it is relegated as a non-important factor.


(4) $$\begin{array}{r} \left(1+2+3+4+5\right)/5=3 \end{array}$$

According to the Pareto principle, the top 20% of the ranking factors determine 80% of the consequences. Thus, the top-ranking 20% are defined as key factors (73). Consequently, this study only compares the differences of the top eight ranking factors between two groups. Ranking difference analysis is just to distinguish the differences between the two groups in the relative importance of factors. In addition, the differences in the absolute importance of perceived factors between the two groups were discussed, and a hypothesis was proposed for testing:

H0: There are no differences between the opinions of groups A and B on the level of importance of factors affecting seismic resilience of RWSSs.

Both the difference of relative importance and absolute importance of factors are analyzed from a local perspective, while Spearman's rank correlation coefficient (ρ) is analyzed from an overall perspective. The purpose of rho test is to confirm whether the grades of all factors perceived by experts from different earthquake-affected areas are consistent under the influence of different factors in rank and importance.

Finally, to reveal the priority of each potential factor when the decision-makers evaluate the earthquake prevention and disaster reduction ability of RWSSs, exploratory factor analysis is used to reduce all factors into a small number of groupings. The reliability and validity of each group were tested by Cronbach's α value and Pearson bivariate correlation analysis (77) since the variance obtained by factor analysis can be used to determine the weight of groups in the construction of composite index (72).

## Results and Discussion

### Descriptive Analysis

Details of these respondents are summarized in Table 3. More than 80% of the respondents had more than 5 years of relevant experience. The respondents were mainly RWSS operation managers (78.8%), and most (76.62%) have experience in earthquake relief. Therefore, most of the respondents in this study have rich experience in the operation and management of RWSSs, and can appropriately represent the opinions from the perspective of RWSS's operation managers. In addition, according to the statistical data in Table 4, there are 94 interviewees in Group A (interviewees had participated in at least one earthquake relief activity of the local RWSS) and 29 interviewees in Group B (interviewees had not experienced earthquake relief activities of the local RWSS).

**Table 3:** Summary of the profiles of respondents.

**Field of work**	**Frequency**	**%**
Designer	7	5.69%
Emergency management officer	9	7.32%
Planner for rural water supply system construction	10	8.13%
Operation management officer	97	78.86%
Experience(years)
<5	23	18.70%
5-10	34	27.64%
10-15	36	29.27%
>15	30	24.39%
Times of participating in earthquake relief of RWSSs
No relevant experience	29	23.58%
1	57	46.34%
2	16	13.01%
≥3	21	17.07%

**Table 4:** Relative importance ranking of influencing factors.

	**Overall (*****n*** **=** **123)**		**Group A (*****n*** **=** **94)**		**Group B (*****n*** **=** **29)**		**Difference between two groups**
	**RII**	**Rank**	**RII**	**Rank**	**RII**	**Rank**	
Leadership	4.492	1	4.517	3	4.436	2	0.081
Alternative water source	4.460	2	4.563	2	4.231	8	0.332
Emergency water supply	4.452	3	4.517	5	4.308	5	0.209
Operation and maintenance funds	4.452	4	4.517	4	4.308	3	0.209
Degree of system recovery	4.444	5	4.414	10	4.513	1	−0.099
Reconstruction model	4.437	6	4.586	1	4.103	20	0.483
Independent Fire-water design	4.429	7	4.506	6	4.256	7	0.250
Topography	4.349	8	4.402	11	4.231	10	0.171
Fast financing access	4.349	9	4.471	7	4.077	25	0.394
Social trust	4.333	10	4.425	9	4.128	18	0.297
Professional reserve	4.325	11	4.356	19	4.256	6	0.100
Organizational structure	4.317	12	4.356	17	4.231	9	0.125
Groundwater stock	4.317	13	4.368	16	4.205	13	0.163
Decision-making	4.310	14	4.368	25	4.308	4	0.060
Earthquake intensity	4.310	15	4.368	15	4.179	17	0.189
Emergency response plan	4.310	16	4.391	12	4.128	18	0.263
Laws and policies	4.286	17	4.379	13	4.077	23	0.302
Remaining service capacity	4.278	18	4.310	25	4.205	15	0.105
Environmental pollution	4.262	19	4.379	14	4.000	28	0.379
Emergency Power	4.262	20	4.345	22	4.077	21	0.268
Crisis insight	4.254	21	4.276	26	4.205	12	0.071
Seismic design	4.246	22	4.345	21	4.026	27	0.319
Post-disaster water demand	4.238	23	4.460	8	3.744	37	0.716
Maintenance information	4.238	24	4.322	23	4.051	26	0.271
Effective partnership	4.230	25	4.241	30	4.205	11	0.036
Earthquake history	4.230	26	4.241	29	4.205	12	0.036
Proactive posture	4.222	27	4.241	28	4.179	14	0.062
Political will	4.175	28	4.356	20	3.769	36	0.587
Intelligent design	4.159	29	4.195	35	4.077	22	0.118
Available financial resources	4.159	30	4.276	27	3.897	34	0.379
Earthquake early warning system	4.143	31	4.218	32	3.974	28	0.244
Climate conditions	4.127	32	4.207	33	3.949	29	0.258
Community participation	4.127	33	4.230	31	3.897	32	0.333
Periodic asset assessment	4.111	34	4.356	18	3.564	41	0.972
The time of the earthquake	4.095	35	4.103	38	4.077	23	0.026
Community publicity	4.056	36	4.126	36	3.897	31	0.229
Household water reserve	4.056	37	4.115	37	3.923	30	0.192
place attachment	4.016	38	4.195	34	3.615	39	0.580
Employment rate	3.881	39	4.011	39	3.590	40	0.421
GRP	3.865	40	3.897	40	3.795	35	0.102
Cultural level	3.825	41	3.874	41	3.718	38	0.156

### Differences in the Importance Ranking of Factors Caused by Earthquake Occurrence

According to the calculated average and SD, all factors are arranged in descending order, which can intuitively compare the differences of the cognition of different respondents on the importance of factors. The mean value, the SD, and ranking of these factors are categorized into three groups from the perspectives of all respondents, namely, Overall, Group A and Group B, as shown in Table 4. Firstly, the mean value of all factors is higher than 3, indicating that all factors are important according to formula 4. Secondly, it is worth noting that except for the degree of system recovery, the respondents who have experienced earthquake disasters scored higher on all other factors than those who have not experienced earthquake disasters, which may indicate that the earthquake made stakeholders assign more emphasis on the impact of factors for system resilience of RWSSs.

In this study, there are 41 factors in total, so the top eight factors of relative importance are identified as the most important top 20%. “Leadership”, “Alternative water source”, “Emergency water supply”, “Operation and maintenance funds,” and “Independent Fire-water design” are the key factors recognized by all respondents of the two groups in this study. Among them, leadership is considered to be the most important factor. Wang shows that in natural crises, leaders play an obviously important role (78). Where leaders take on a moral leadership approach (an egalitarian leadership style in which leaders lead by example in the disaster relief effort), this results in higher quality decision-making than occurs with authoritarian leadership, the failure of which can have drastic consequences. Examples of the consequences of poor leadership management are revealed in the Katrina Crisis (79) and in post-disaster recovery (80). In rural areas of China, due to the challenges of both catastrophe scenarios and the institutional environment, strengthening the leadership construction of grassroots leaders will effectively enhance the local emergency response capacity (81).

Bruneau et al. describe system resilience as comprising robustness, redundancy, resource access, and response rapidity with the redundant design of WSSs (15), along with alternative water sources as being the important factor affecting the system resilience (15, 25). In any event, the responsible body that operates the water supply should ensure that the water supply service is not interrupted (7). That is, people in a disaster area must be provided with an emergency water supply through water delivery vehicles, fire engines, or the laying of temporary pipelines. The interruption of water supply after an earthquake can prompt secondary disasters, such as fire, as exemplified by the 1995 Hanshin (Kobe), Japan (25). Therefore, independent fire water supply design is critical. Consequently, the Chinese government issued a special policy to discuss the selection criteria of emergency water sources after the Wenchuan earthquake (82). The poor operation of Kathmandu WSS led to an under capacity to cope with the aftermath of this earthquake. This failure was attributed to a lack of operation and maintenance funds, technical personnel, and system information (35). Indeed, such weaknesses will be more serious in rural areas because water infrastructure, operation, maintenance, and financial sustainability may be suboptimal (1).

The “reconstruction model” was considered as the most important factor by the respondents from Group A. Internationally, reconstruction models are generally divided into donor-driven reconstruction and owner-driven reconstruction. Traditionally, a donation-driven approach is generally considered to be a more suitable model for reconstruction. However, some research has indicated that the owner-driven model is preferable (65), especially when considering long-term disaster resilience (66). There is a growing consensus regarding the link between post-disaster reconstruction and disaster resilience (83). In addition, “fast financing access” (ranked 7 in group A) and “post-disaster water demand” (ranked 8 in Group A) were also considered key factors by respondents who had experienced an earthquake, which is consistent with the research conclusion of UWSS system in literature (15, 25). In the aftermath of an earthquake, people may migrate from seriously damaged areas to safe areas, such as temporary evacuation points, and consequently, water demand will also change. In the most extreme cases, where the WSS is completely destroyed, there may be no adverse consequences since there may be no water demand once people have evacuated. Contrariwise, even where the WSS is undamaged, the increased population at temporary gathering points and other migration areas may be unable to meet the heightened levels of water demand in the short-term (35). Therefore, it is critical to forecast the post-disaster water demand at different locations.

“Degree of system recovery” was ranked as the most important factor by respondents from group B. Apart from the immediate effects of the destruction, disasters present an opportunity to improve the living conditions of those living within a disaster risk area through effective and resilient reconstruction (84). In other words, reconstruction is an opportunity to strengthen the future resilience of a community (66). “Decision-making “and “Professional reserve” were also considered as key factors. Decision-making is defined as clear authorization, which enables highly skilled workers to make appropriate decisions in response to disasters, which is an important factor affecting the organizational resilience of WSSs (7). Due to low population density, large service areas, and income constraints, the operation, maintenance, and financial sustainability of RWSSs are typically suboptimal (1). Moreover, RWSSs tend to lack professional staff. This shows that the respondents from group B have a lower expectation of earthquake response, and thus pay greater attention to the ability of RWSSs to maintain normal operation. This is because the three factors directly affect the ability of RWSSs to resist the interference of manmade or natural disasters, while the ones from group A are more concerned about some specific factors affecting the ability to recover from an earthquake.

To compare whether there was a significant difference between the two groups, a *T*-test or non-parametric Mann–Whitney *U*-test was used, depending on whether the data were normally distributed, with *p* < 0.05 as the level of statistical significance. According to statistical analysis, there were significant differences in Wallis values of 10 factors between the two groups (*p* < 0.05), and the significance level was shown in Table 5. It indicates that there are differences in direct experience with earthquakes in the importance of specific factors. However, in general, the hypothesis is validated as true, where 31 out of 41 factors are validated without a significant difference in importance between two groups.

**Table 5:** Significance test results of Group A and Group B.

**No**.	**Factors**	**Kolmogorov–Smirnov**	**Mann–Whitney *U*-test**
		***P*** **-value**	***P*** **-value**
SF02	Maintenance information	0.000	0.032
GF17	Place attachment	0.000	0.001
SF13	Employment rate	0.000	0.006
SF06	Periodic asset assessment	0.000	0.044
GF10	Community participation	0.000	0.014
GF18	Social trust	0.000	0.041
GF25	Reconstruction model	0.000	0.002
GF21	Available financial resources	0.000	0.029
GF22	Fast financing access	0.000	0.004
SF15	Environmental pollution	0.000	0.011

The ranking difference analysis of the above key factors and the importance comparison of each were analyzed to determine whether the occurrence of an earthquake affects the perception of stakeholders on the importance of these factors, as seen from the local perspective. Spearman's correlation coefficient ranking method was used to test the consistency of all respondents on factor importance ranking. As it turned out, the importance ranking of the influencing factors is broadly and highly consistent and significant between the two groups (rho > 0.5, *p* < 0.5), which is consistent with the conclusion of the previous analysis. Even so, certain local differences are evident.

### The Important Factors of Seismic-Resilience for RWSSs

The first step in factor reliability analysis is to estimate the sample size (85). To satisfy the ratio of the sample size to the number of variables (5.00) recommended by Bentler and Chou (86), the overall factor analysis of 41 influencing factors cannot be carried out, but must be conducted into two steps: GFs (including 26 factors) and SFs (including 15 factors). As the ratio of GFs is slightly less than 5.00, several factors will later be deleted according to the loading value of less than 0.5 after rotation. To further the data suitability for the analysis, the Kaiser–Meyer–Olkin (KMO) measure of sampling adequacy and Bartlett's test of Sphericity were used to test the applicability of the data. Both KMO values of GFs (0.892) and SFs (0.882) are higher than the recommended threshold of 0.6, indicating that the degree of common variance among factors is high. Meanwhile, the value of Barrett's test is also large (1850.655 and 948.262) and significant (0.000 <0.05), indicating that the data obtained is suitable for factor analysis.

**Figure 4:**
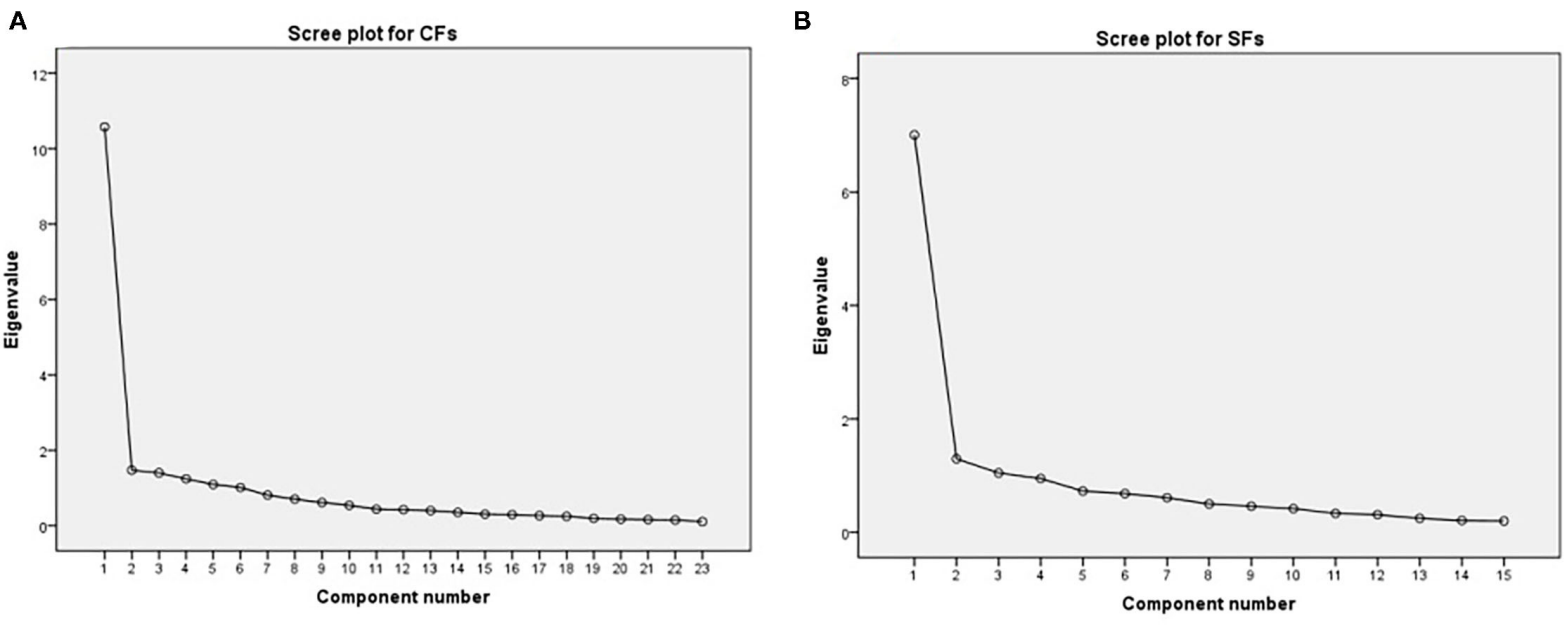
Scree plot of factors affecting the seismic resilience of RWSSs. **(A)** CFs, **(B)** SFs.

In order to determine the minimum number of components representing the relationship between a group of variables, principal component analysis was used to extract factors. Kaiser criterion was used in this study, and only the factors with eigenvalues of 1.0 or above were retained. In this study, the most commonly used maximum variance method is used, in which a load of each factor in each component is set to a conventional high value of 0.5. Additionally, to meet the ratio of sample size, four factors with loading less than 0.5 are deleted (independent fire-water design, earthquake early warning system, organization structure, and household water reserve), leaving the ratio of GFs at 5.35 and the ratio of SFs at 8.79, both of which are greater than 5. Table 6 shows the factor groupings based on maximum variance rotation. A total of nine factor groups were extracted through principal component analysis, of which the explanation rate of six factor groupings for GFs was 73.036%, with 62.321% for three factors groupings for SFs (see Figure 4), which is higher than the recommended 60% (73).

**Table 6:** Rotated component matrix.

	**GFs**							**SFs**		
**Factor**	**1**	**2**	**3**	**4**	**5**	**6**	**Factor**	**7**	**8**	**9**
GF25	0.726						SF02	0.711		
GF20	0.725						SF01	0.763		
GF23	0.689						SF04	0.762		
GF09	0.708						SF12	0.696		
GF21	0.650						SF08	0.637		
GF22	0.634						SF05	0.583		
GF10	0.560						SF06	0.539		
GF17		0.750					SF15		0.837	
GF18		0.691					SF09		0.650	
GF08		0.675					SF07		0.599	
GF06			0.698				SF10		0.599	
GF14			0.692				SF14			0.766
GF12			0.602				SF13			0.747
GF19			0.656				SF11			0.734
GF24				0.832						
GF26				0.739						
GF11				0.507						
GF07					0.779					
GF16					0.597					
GF13					0.594					
GF03						0.788				
GF02						0.736				
GF01						0.732				
Eigenvalue	10.575	1.473	1.401	1.242	1.094	1.013	7.005	1.297	1.046
Variance (%)	45.979	6.402	6.091	5.400	4.758	4.405	46.700	8.646	6.975
Cumulative	45.979	52.382	58.472	63.872	68.630	73.036	46.700	55.346	62.321
Cronbach's α	0.904	0.860	0.771	0.812	0.802	0.803	0.881	0.824	0.801

*Rotation converged in 8 and 6 iterations, respectively. Extraction method: principal component. Rotation method: Maximum variance method*.

Considering the three stages of earthquake disaster prevention, emergency response, and post-disaster recovery, and in accordance with established research on the internal relationship between heavy load factors and grouping factors (77), the nine groups are identified as follows: G1 “Economic resilience”; G2 “Social resilience in the disaster prevention stage”; G3 “Adaptive capacity in the emergency response stage”; G4 “Environmental vulnerability in the disaster prevention stage”; G5 “Restorative capacity in the post-disaster recovery stage”; G6 “Technical resilience in the disaster prevention stage”; S1 “Organizational resilience in the disaster prevention stage”; S2 “Environmental resilience in the disaster prevention stage”; and S3 “Economic condition in the disaster prevention stage.” Cronbach's α value of each group is calculated based on the aggregation factor in each group, as shown in Table 6. All Cronbach α values (0.771 – 0.904) were greater than the critical value of 0.7, indicating that all the groups are reliable. The Pearson bivariate correlation analysis result shows that the correlation coefficient among the factors is high and significant, which indicates that each group can be measured by cluster factor. Considering the space constraints, only the correlations between G1 “Economic resilience” factors are listed in Table 7; thus, the validity of the groups is also verified.

**Table 7:** The Pearson correlations among factors in G1.

**Factors**	**GF25**	**GF20**	**GF22**	**GF21**	**GF10**	**GF23**	**GF09**
GF25-Reconstruction mode	1	0.547^**^	0.598^**^	0.569^**^	0.600^**^	0.537^**^	0.573^**^
GF20- Operation and maintenance funds	0.547^**^	1	0.563^**^	0.620^**^	0.493^**^	0.538^**^	0.578^**^
GF22-Fast financing access	0.598^**^	0.563^**^	1	0.664^**^	0.581^**^	0.616^**^	0.503^**^
GF21-Available financial resources	0.569^**^	0.620^**^	0.664^**^	1	0.609^**^	0.605^**^	0.574^**^
GF10-Community participation	0.600^**^	0.493^**^	0.581^**^	0.609^**^	1	0.550^**^	0.487^**^
GF23-Earthquake intensity	0.537^**^	0.538^**^	0.616^**^	0.605^**^	0.550^**^	1	0.636^**^
GF09-ERP	0.573^**^	0.578^**^	0.503^**^	0.574^**^	0.487^**^	0.636^**^	1

** *Correlation is significant at the 0.01 level (two-tailed)*.

G1— Economic resilience. Economic resilience is an important part of the seismic resilience of WSS (15, 25, 35, 39), which affects other factors indirectly and directly (39). This factor grouping includes seven factors, among which the reconstruction model is considered to be the most important economic driving factor for the seismic resilience of RWSSs. Due to the great threat of earthquakes, the Chinese government has been exploring post-disaster reconstruction models. Different reconstruction models mean different financial allocations. For example, the reconstruction cost of Wenchuan was mainly allocated by the central government since the reconstruction was led by the state, while the Sichuan provincial government was mainly responsible for the funds of Lushan post-earthquake reconstruction. The local government of Aba was mainly responsible for the funds for post-earthquake reconstruction of Jiuzhaigou. In addition to financial allocation, catastrophe insurance, loans, counterpart assistance, and social participation (social donation) are important sources of funds for post-disaster reconstruction of WSS. Rapid access to these post-disaster reconstruction funds directly affects the recovery speed (15, 25, 39). Besides, sufficient operation and maintenance funds help to improve the anti-interference ability of RWSSs during the disaster prevention stage (35). An emergency response plan can also improve the resilience of the system by providing employees with necessary seismic training and awareness (7). In addition, the past experience of earthquake disasters reveals that earthquake intensity strongly impacts economic resilience. Different types of earthquake damage draw on different funds for post-disaster recovery and reconstruction. For highly damaging earthquakes, in addition to national and local government grants, international assistance is even sometimes required (25), such as in the case of the Nepal earthquake (35).

G2— Social resilience in the disaster prevention stage. This factor group includes three factors: place attachment, social trust, and topography. People's sense of belonging to their place of residence and their trust in the local government and military support during a disaster make them more willing to participate in local earthquake relief and post-disaster reconstruction activities. Similarly, as the latest research of Ao et al. on the flood resistance capacity of rural areas emphasizes, strengthening the trust of rural residents in the government's flood control capacity in the disaster prevention stage is necessary in order to effectively improve social disaster prevention capacity (62). Resilience has spatial differences. The resilience of most urban areas is higher than that of rural areas (42, 87), and according to the research of Sung and Liaw in Taiwan, topography is the most important factor causing social and economic differences as the socio-economic resilience of mountainous areas is often relatively low (87). Compared with urban areas, the topography in rural areas of China is complex and changeable. Most water supply pipelines of UWSSs are mainly buried pipelines. However, in rural areas, due to topography constraints, some pipelines are suspended on steep slopes, facing the risk of landslide and mountain flood disasters, affecting the system resilience in the disaster prevention stage.

G3— Adaptive capacity in the emergency response stage. This group is composed of four highly relevant factors: remaining service capacity, water demand after the disaster, emergency water supply, and leadership. The absorptive capacity refers to the ability of the system to absorb and minimize the consequences of the shock with an acceptable amount of effort (25). It is related to the functionality after interruption (14). It is, therefore, no surprise that the remaining service capacity of the system has the highest correlation with G3. Adaptive capacity is defined as the ability of the system to adjust to the undesirable consequences of external shock (25). When water demand is higher than the remaining water supply capacity of the system itself, the emergency water supply can make up for the shortage, with the leadership of decision-makers impacting the adequate supply of water in the emergency response stage.

G4— Environmental vulnerability. This group consists of three factors: earthquake history, the time of the earthquake, and effective partnership. Among which the largest load is earthquake history. Mostafavi et al. studied the earthquake history of the Kathmandu Valley area and found that destructive earthquakes would inevitably occur in the area (35). Many studies have shown that the earthquake may change the local geological environment, thus triggering a variety of geological disasters and aggravating the vulnerability of the environment (46, 64), particularly, destructive earthquakes, such as the Wenchuan earthquake in 2008. In addition, effective partnerships are also considered to be important factors affecting resilience (7). Maintaining a good cooperative relationship with partners during the disaster prevention stage can ensure that the water supply organization can seek resources and help from partners after the earthquake, so as to improve the seismic resilience of the WSS. The water supply capacity of WSSs in the post-earthquake period depends to a certain extent on the timing of the earthquake. Earthquakes occurring during the night or early morning hours may cause more serious consequences since disaster mitigation efforts at night are more difficult to carry out (67).

G5— Restorative capacity in the post-disaster recovery stage. This group includes three factors, among which the load of degree of system recovery is the largest. The restorative ability of the system can be expressed by the recovery speed, where the faster the system recovers to an acceptable level, the higher the recovery ability will be (15, 25). Different systems have different requirements on the degree of recovery. For limited infrastructure hardening, they usually need to recover to greater than 100% of the pre-earthquake level (15). Due to the casualties and infrastructure damage caused by the earthquake, there are potential crises, such as the fire experienced after the Kobe Earthquake of 1995 (60), cholera after the Haiti Earthquake of 2010 (88), and the riots after the Chile Earthquake of 1960 (37), which all affect system recovery. The higher the insight into these crises, the lower the probability of secondary damage to the system, and the higher the recovery ability of the system. Besides, the decision-making of stakeholders also affects the post-disaster recovery capacity of the system (7).

G6— Technical resilience in the disaster prevention stage. This group includes three highly relevant technical design factors: emergency power, seismic design, and alternative water source. The physical vulnerability and subsequent restoration of WSSs, including pipe networks and water sources, refer to the technical dimension of resilience, which has been the core theme of research regarding the seismic resilience of WSSs (25, 61). Although physical strengthening can improve the seismic resilience of the system, if the capital budget, geographical environment constraints, and later operation and maintenance support are not considered, this resilience strengthening may be ineffective, especially in rural areas with relatively poor economic and environmental conditions (37).

S1— Organizational resilience in the disaster prevention stage. Organizational resilience is considered to be a key dimension for evaluating the resilience of WSS (7, 15, 25, 38). There are seven factors in this factor grouping: political will, professional reserve, maintenance information, periodic asset assessment, law and policy, proactive posture, and community publicity. Social resilience to disasters depends largely on the political commitment to building resilience through the allocation of resources, such as investment in early warning systems, disaster vulnerability reduction activities, etc. (89). Thus, the political will of decision-makers is considered to be an important organizational factor since it affects the decision-making and implementation process (7). However, few pieces of research have explored the impact of political will on rural drinking water safety. The research on water supply safety in rural areas of Canada shows that if the complex economic and social factors in rural areas are not considered, political commitment may bring a burden to rural drinking water safety (51). Maintenance records and sufficient professional reserves can effectively shorten the post-disaster recovery period (35). However, in rural areas, there is a lack of professionally trained personnel. Firstly, the wage level and career development opportunities in rural areas are much lower than those in urban areas, and it is difficult to attract professionals. Secondly, the rural terrain is complex and vast, and non-local personnel are usually unable to eliminate pipe network faults in time and effectively. At the same time, the educational level of rural residents is generally relatively low. The training of rural residents will also lead to new problems such as time and cost, which will threaten the drinking water safety of RWSSs (51). In addition, in this study, laws, policies, and proactive emergency drills are also considered to be important influencing factors affecting organizational resilience by stakeholders of RWSSs, which is consistent with the research conclusion of UWSSs (7). Regular assessment of the asset life of the WSS and planning and implementation of repair or replacement investment before failure also contribute to the resistance of the system to earthquake disasters (57). Community publicity and proactive posture are also important measures in improving organizational resilience in the disaster prevention stage. Proactive posture was one of the more important indices in evaluating the organizational resilience of WSSs (7). In addition, research by Ao et al. on the hardest-hit areas in Wenchuan also confirmed that extensive disaster publicity in rural areas was an effective means for improving the disaster prevention capacity at the disaster prevention stage (90).

S2— Environmental resilience in the disaster prevention stage. This group consists of four factors: groundwater stock, environmental pollution, climate conditions, and household water reserve. Recent studies have shown that the environmental dimension is the important influencing factor of resilience that has been ignored for a long time (23, 25). Groundwater stock and environmental pollution directly affect the water source safety of RWSSs. In April 2015, the State Council issued the “action plan for prevention and control of water pollution” in order to improve the water environment (91). This is a guide on the national water pollution prevention and control of China for the years 2015 to 2030. In China, household water reserves, such as wells, only exist in rural areas, which can alleviate the water demand after an earthquake to a certain extent, such as in Kathmandu. However, the water quality of household water sources is not guaranteed. Moreover, the existence of household water sources may endanger the stock of local groundwater (35). Climate conditions will also affect the seismic resilience of the WSS. Generally speaking, the water shortage caused by earthquakes in summer is more serious than those in winter (25, 35). Moreover, for areas with tropical or subtropical climates, the water shortage caused by earthquakes in summer may aggravate further disasters arising from the urban heat island phenomenon (92).

S3— Socio-economic condition at the disaster prevention stage. This factor group is mainly related to Gross Regional Product, cultural level, and employment rate. Employment rate of local residents and GRP are usually used to measure economic resilience (15, 25, 57). Generally, the area with higher level has higher resilience (25, 39). In addition, the cultural level is also considered to be one of the driving factors of resilience (64), In China, the educational level of rural residents is generally low, which affect their employment and income level to a certain extent.

## Conclusion

Rural water supply systems (RWSSs) are an important class of infrastructure supporting rural development and prosperity. In order to improve the reliability of RWSSs, it is necessary to clearly understand the factors influencing the seismic resilience of RWSSs. This study attempts to identify the factors impacting the robustness of the seismic resilience of RWSSs and to determine the key influencing factors from the perspective of stakeholders. First, a list of 41 factors affecting system resilience was obtained through a comprehensive literature review. This was followed by the semi-structured expert interview to test the validity of the extracted factors for the context of RWSSs. Out of this, a questionnaire was developed to investigate the views of stakeholders of RWSSs on the importance of these 41 factors. According to the results from 123 valid questionnaires, the mean and SD of 41 factors were calculated and sorted. It was found that earthquake experience affects the views of stakeholders on the importance of certain factors. Finally, 41 factor groups were reduced to 9 factor groups through a two-factor analysis. According to the results of factor analysis of GFSs, “economic resilience” is considered to be the most important factor grouping of GFs by stakeholders of RWSSs. This was followed by “social resilience in the disaster prevention stage,” “adaptive capacity in the emergency response stage,” “environmental vulnerability,” “restorative capacity in the post-disaster recovery stage,” and finally, “technical resilience in the disaster prevention stage.” The factor analysis results of SFs showed that “organizational resilience in the disaster prevention stage” is considered to be the most important factor group of SFs by stakeholders of RWSSs. This was followed by “environmental resilience in the disaster prevention stage” and “socio-economic condition at the disaster prevention stage.”

As a result of this study, several implications can be drawn, as follows.

For the seismic resilient construction of RWSSs, the improvement of “soft” resilience (as distinct from technical hard resilience) of organizations, society, and the environment is emphasized. This stands in contrast to UWSS strengthening as described in the bulk of previous research, which prioritizes technical resilience of the system. Compared with UWSSs, the financial resources for the operation and maintenance of RWSSs may be suboptimal (1), meaning that decision-makers operating under the constraints of limited economic resources should give priority to “soft” resilience factors as described here. Technical strengthening measures can continue to be considered when carrying out new construction or post-disaster reconstruction systems that increase standby water sources to the extent that finances and budgeting allow.

It is not appropriate to use fixed weights when evaluating the seismic resilience of RWSSs given the variability in multi-criteria decision-making models used in seismic resilience of RWSSs. The seismic resilience of RWSS is affected by multi-dimensional factors. It is found that the decision-makers experienced with earthquakes are more concerned with the sources of reconstruction funds and water demand after the disaster, while those decision-makers who have never experienced an earthquake are more concerned with the ability of the system to resist external interference in the disaster prevention stage. This study reveals that the decision-makers of different regions have different priorities. Thus, in developing goals related to seismic resilience construction of local RWSS, there may be different resilience targets established across different regions. Largely, this is because earthquake events do not occur evenly in every region, nor do they occur periodically. Therefore, regional considerations and conditions need to be taken into account when developing a seismic resilience evaluation model of RWSSs. This can be done by assigning appropriate weights that reflect local conditions and risk priorities to indicators.

The exploration of factors affecting the seismic-resilience of RWSSs contributes to the body knowledge on resilience of WSSs by identifying relevant factors and revealing the influence that spatial differences bring to a cognitive assessment of their importance as they pertain to seismic-resilience of RWSSs. This understanding helps the various stakeholders to better implement the resilience practices of RWSSs. This is especially true of those managers of RWSSs located proximate to seismic zones but who have not yet experienced a destructive earthquake.

This study is also relevant for other developing countries, apart from China, that suffer frequent earthquake disasters. Local managers of RWSSs can refer to the seismic resilience factors list of RWSSs given in this study and adjust and weigh the factors in combination with the actual situation of local WSSs they experience on the ground in order to evaluate the seismic resilience of their own RWSSs.

However, this study is not without limitations. Due to the constraints of sample size, factor analysis had to be conducted in two parts in order to satisfy reliability requirements, reducing the certainty of the relationship between GFs and SFs. This, however, was not the focus of this study. In future research, it is planned to apply structural equation modeling to fully address and determine the relationship between various factor groups.

## Data Availability Statement

The raw data supporting the conclusions of this article will be made available by the authors, without undue reservation.

## Ethics Statement

Written informed consent was obtained from the individual(s) for the publication of any potentially identifiable images or data included in this article.

## Author Contributions

WZ and GK were responsible for the conception and design of the article, data analysis, and thesis writing. WZ and CC were responsible for the implementation of the research, data analysis, and interpretation of the results. IM was responsible for the quality control and the overall review of the article. All authors contributed to the article and approved the submitted version.

## Funding

This study was supported by the National Natural Science Foundation of China (No. 71971147). The funders have no role in the study design, analysis, and interpretation of the study findings.

## Conflict of Interest

The authors declare that the research was conducted in the absence of any commercial or financial relationships that could be construed as a potential conflict of interest.

## Publisher's Note

All claims expressed in this article are solely those of the authors and do not necessarily represent those of their affiliated organizations, or those of the publisher, the editors and the reviewers. Any product that may be evaluated in this article, or claim that may be made by its manufacturer, is not guaranteed or endorsed by the publisher.
